# Autoantibodies in the diagnostics, prognostics and follow-up of primary biliary cholangitis

**DOI:** 10.1016/j.jtauto.2026.100366

**Published:** 2026-03-19

**Authors:** Péter Antal-Szalmás, Dóra Bencze, Sarolta Demeter, Krisztina Pénzes-Daku, Lilla Szabó, Beáta Tóth, Róza Földesi, Mária Papp, Gábor Nagy

**Affiliations:** a Department of Laboratory Medicine, Faculty of Medicine, University of Debrecen, Debrecen, Hungary; b Doctoral School of Medical Sciences, University of Debrecen, Debrecen, Hungary; c Department of Gastroenterology, Faculty of Medicine, University of Debrecen, Debrecen, Hungary

**Keywords:** Autoimmune liver disease, Primary biliary cholangitis, Anti-mitochondrial antibody, Anti-gp210, Anti-sp100, Anti-HK1, Anti-KLHL12

## Abstract

Primary biliary cholangitis (PBC), is a chronic autoimmune liver disease, characterized by cholangiopathy, cholestasis and in the long-term fibrosis, biliary cirrhosis and ultimately end-stage liver disease unless liver transplantation is applied. The diagnostics of PBC is based on biochemical tests, autoantibody measurements and liver histopathology. Abdominal ultrasound, later magnetic resonance cholangiopancreatography (MRCP) or endoscopic ultrasound can be effective methods for imaging the intra- and extrahepatic bile ducts. Concerning autoantibodies, anti-mitochondrial (AMA) and PBC-specific antinuclear antibodies (ANA) are the front-line tests that can be identified by indirect immunofluorescence tests or solid-phase immunoassays. AMA and especially the AMA-M2 variant have a high sensitivity and specificity for PBC, while anti-gp210 and anti-sp100 (PBC-specific ANAs) have a lower sensitivity but very high specificity for the disease. Anti-centromere antibodies (ACA) can help in the diagnostics of PBC and some overlap syndromes. Novel emerging markers – anti-hexokinase 1 and anti-Kelch-like 12 protein (anti-KLHL12) – can help the identification of rare AMA- and ANA-negative PBC cases. There is cumulating evidence that beside diagnostics certain autoantibodies can provide information about the prognostics of PBC, can predict therapy response, furthermore, can act as activity marker in follow-up of the patients during therapy. We summarize here the most important data about the accepted and potential clinical applications of traditional and emerging new autoantibodies in PBC.

## 1 Introduction

Primary biliary cholangitis (PBC), (formerly primary biliary cirrhosis), is an autoimmune, chronic cholangiopathy, characterized by the progressive destruction of biliary epithelial cells and small, intrahepatic bile ducts. This leads to cholestasis and in the long term to fibrosis, biliary cirrhosis, and ultimately end-stage liver disease unless liver transplantation is applied.

The shift in terminology from primary biliary cirrhosis to primary biliary cholangitis reflects a substantial change in the natural history and clinical presentation of the disease over recent decades [1]. In the pre-antimitochondrial antibody (AMA) era, diagnosis was frequently established at an advanced stage, and in several historical cohorts substantial proportion of patients already exhibited histological or clinical evidence of cirrhosis at the time of recognition [2]. The introduction of AMA testing enabled much earlier identification, often years before symptom onset; AMA positivity may precede clinical PBC by a median of approximately 6 years (range 1–19 years), although only a minority of individuals with isolated AMA positivity ultimately develop overt disease [3]. Besides early and efficient diagnostics, the introduction of ursodeoxycholic acid (UDCA) has further reduced or decelerated the rate of cirrhosis development in PBC patients. Before UDCA therapy, the natural history was characterized by a 10-year survival of 50–70% in asymptomatic patients and a median survival of 5–8 years in symptomatic individuals [3,4]. In the contemporary era, the majority of patients achieve at least a partial biochemical response to UDCA, while approximately 25–30% attain complete biochemical normalization. Overall, in modern cohorts with long-term follow-up, progression to cirrhosis is observed only in approximately 15–20% of patients [5]. Accordingly, the term “cholangitis” more accurately reflects both the underlying immune-mediated cholangiopathy and the markedly improved prognosis in the era of early serological diagnosis and effective therapy.

PBC predominantly effects women over the age of 40, and its incidence in the Asian-Pacific region is about 8.4, in Europe 18.6 and in North America 27.5 per 1,000,000 inhabitants per year. Concerning the prevalence, it is 108.5, 145.9 and 218.1 per million in the above-mentioned regions, respectively. Pruritus, fatigue, sicca complex and abdominal discomfort are the leading complaints of these patients, that are associated with signs and symptoms of cholestasis and liver destruction. Rare manifestations are restless legs, sleeplessness, psychological and cognitive dysfunctions [[6], [7], [8], [9], [10], [11], [12]]. Several extrahepatic autoimmune diseases could coexist with PBC, such as Sjögren's syndrome (3.5–73%), autoimmune thyroid disease (5.6–23.6%), systemic sclerosis (1.4–12.3%), Raynaud's phenomenon (1.8–5.6%), systemic lupus erythematosus (0–3.7%), and celiac disease (0–6%) [13,14]. The diagnostics of PBC is based on biochemical tests, PBC-specific autoantibodies and histological evaluation of biopsy samples. Though the importance of autoantibodies is well-known in diagnostics of PBC and they are incorporated into current international guidelines, too, but data about the other possible ways of clinical application – presented in Table 1 – is less characterized and will be discussed in detail here.

**Table 1:** Autoantibodies used in the diagnostics, prognostics and follow-up of patients with PBC.

Antibody	Diagnosis	Prognostics	Activity	Follow-up	Special cases
**AMA-M2**	+++	+	++	+	AMA+/PBC-
					AMA-/PBC+
**anti-gp210**	+++	+++	++	+	
**anti-sp100,anti-PML**	+++	+++	++	+	
**ACA**	+	++	-	ND	PBC + SSc
					PBC + pSS
**anti-HK1**	++	+	+	+	
**anti-KLHL12**	++	-	+	-	

ACA: anti-centromere antibodies; ΑΜΑ-M2: anti-mitochondrial M2 group antibodies; HK-1: hexokinase 1; KLHL12: kelch-like 12 protein; PML: promyelocytic leukemia proteins; sp100: nuclear body speckled 100 kDa; pSS: primary Sjögren's syndrome; SSc: systemic sclerosis; AMA+/PBC-: AMA-positive, no signs and negative biochemical markers of PBC; AMA-/PBC+: AMA-negative, signs and positive biochemical markers of PBC; PBC + SSc: PBC and systemic sclerosis overlap; PBC + pSS: PBC and Sjögren's syndrome overlap.

+++: included in guidelines; ++: clear evidence; +: some supporting data are available; -: no correlation; ND: no data.

## 2 Diagnostic autoantibodies in PBC

### 2.1 Anti-mitochondrial autoantibodies as diagnostic markers

Anti-mitochondrial antibodies (AMA) are sensitive, but less specific markers of PBC. The most important target antigen is the E2 subunit of the pyruvate-dehydrogenase complex (E2-PDC). About 95% of PBC patients show reactivity to PDC-E2 and 50%-70% towards 2-oxoglutarate dehydrogenase complex (OGDC) and branched-chain 2-oxo acid dehydrogenase complex (BCOADC). This antigen group is called AMA-M2, that can be identified by immunoblot or solid phase immunoassays [8,15].

### 2.2 Anti-nuclear autoantibodies as diagnostic markers

Anti-nuclear autoantibodies (ANA) can be present in about 30-50% of patients with PBC. The multiple nuclear dots pattern (MND, AC-12) caused by antibodies specific for speckled protein 100 kDa (sp100), promyelocytic leukemia protein (PML), speckled protein 140 kDa (sp-140) and/or small ubiquitin-like modifier (SUMO) that are components of the PML nuclear bodies are rather specific for PBC (>95%). The rate of positivity is 8-44% and 12-22% for anti-sp100 and for anti-PML, respectively, and they appear together frequently. The pooled sensitivity and specificity of anti-sp100 for PBC is 23.1% and 97.7% [8,[16], [17], [18]].

The punctate nuclear envelope pattern is caused dominantly by the anti-gp210 (glycoprotein 210 kDa) antibodies and much rarely by anti-lamin B receptor and/or anti-nucleoporin p62 targeting components of the nuclear pore complex. The sensitivity and specificity of anti-gp210 for PBC is in the range of 5.7-55.9% and 61.7-100%, respectively [8,19,20].

Anti-centromere autoantibodies (ACA) target the centromere-kinetochore macromolecular complex and dominantly associated with systemic sclerosis (SSc) but can be present in 9-30% of PBC cases, and in 80% of the PBC/SSc overlap syndrome [8,21,22].

### 2.3 Novel autoantibodies as diagnostic markers

Further autoantibodies frequently associated with PBC are anti-hexokinase 1 (anti-HK1) and anti-Kelch-like 12 protein (anti-KLHL12). Anti-HK1 targets the enzyme HK1, localized in the outer membrane of mitochondria, that is involved in glucose metabolism and apoptosis. The autoantigen for anti-KLHL12 is Kelch-like 12 that is a collagen exporter and plays a role in ubiquitination. Anti-HK1 and anti-KLHL12 are highly specific for PBC (>95%) [8], while a recent study of Reig et al. showed that 46.1% and 22.8% from 254 PBC patients were anti-HK1 and anti-KLHL12 positive, respectively [23]. It is even more important that these antibodies can be present in those cases which are negative for AMA and/or PBC-specific ANAs. The introduction of anti-HK1 and anti-KLHL12 enhanced the diagnostic sensitivity of AMA-negative PBC to 68.5% - 75%, depending of the method used for antibody detection [8,24]. In another study anti-HK1 and anti-KLHL12 were present in about 40% of AMA- and/or PBC-specific ANA-negative patients [8,23].

Anti-translocator promoter region (Anti-Trp) antibodies are novel markers of PBC, requiring additional research [25]. The presence of Anti-Saccharomyces antibody (ASCA) in PBC was about 20-30% and was associated with elevated levels of the cholestatic biochemical markers [26]. Rarely (up to 30%), antibodies against the extractable nuclear antigens (anti-RNP, anti-Scl70, anti-Jo1) can be also detected in the serum of PBC patients with a coexisting systemic autoimmune disorder [27,28].

### 2.4 Current guidelines about the diagnostics of PBC

The most important diagnostic markers presented in the recommendations and supporting text of the PBC guidelines prepared by 3 major scientific societies – European Association for the Study of the Liver (EASL), American Association for the Study of Liver Diseases (AASLD) and Asian Pacific Association for the Study of the Liver (APASL) – are presented in Supplementary Table 1. According to these the diagnostics of PBC is based on serum biochemistry tests, autoantibody determinations, imaging examinations and in certain cases liver biopsy. The diagnostic criteria for PBC is: i) serum cholestatic markers (dominantly alkaline phosphatase - ALP) are elevated; ii) AMA is present in >1:40 titer or PBC-specific ANA (AC-6, multiple nuclear dots - MND, anti-sp100 antibodies or AC-12, punctate nuclear envelope, anti-gp210 antibodies) is detected; iii) liver histology is showing nonsuppurative destructive cholangitis involving the interlobular bile ducts. If two out of these 3 criteria are present the diagnosis of PBC is solid [6,10,[29], [30], [31]]. Though ALP is used most widely in diagnostics of PBC, it should be evaluated carefully since several extra-liver sources of it exist, such as bones, intestines, placenta and some tumors [10].

**Fig. 1:**
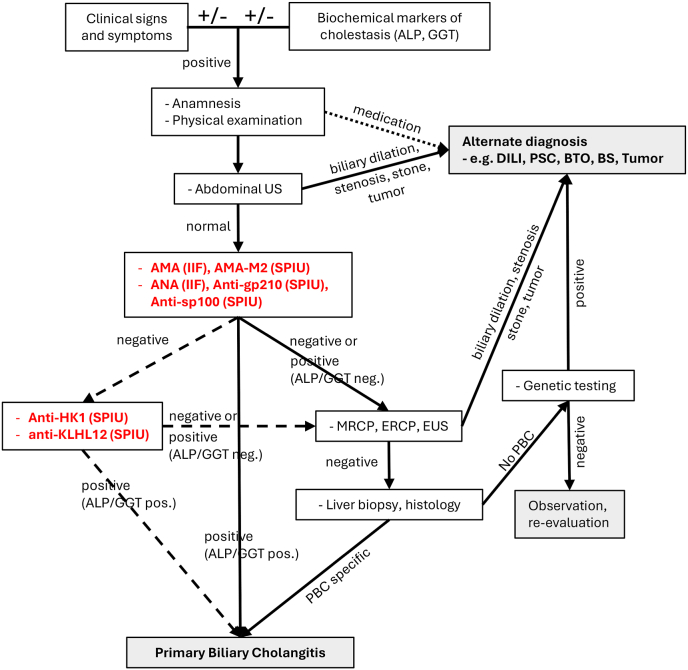
Diagnostic algorithm of PBC. The diagnostic criteria for PBC are: i) serum cholestatic markers (dominantly alkaline phosphatase) are elevated; ii) AMA or PBC-specific ANA is present; iii) liver histology is showing nonsuppurative destructive cholangitis involving the interlobular bile ducts. If two out of these 3 criteria are present the diagnosis of PBC is solid. Furthermore, imaging techniques can support diagnostics. In AMA-negative/ANA-negative cases anti-HK1 and anti-KLHL12 can be evaluated for identification of cholestatic patients with PBC. If the signs and symptoms are typical for PBC but the biochemical or available serological markers are negative, furthermore the imaging technics do not identify other liver disorders histological evaluation of liver biopsy sample can decide. When both the biochemical and serological tests are negative individual decision is required concerning liver biopsy. ACA: anti-centromere antibody, ALP: alkaline phosphatase, ALP/GGT pos.: if ALP and/or GGT is positive, ALP/GGT neg.: if ALP and GGT is negative, AMA: anti-mitochondrial antibody, ANA: anti-nuclear antibody, anti-HK1: anti-hexokinase 1, anti-KLHL12: anti-Kelch-like 12 protein, anti-PML: anti-promyelocytic Leukemia protein, BS: biliary stone, BTO: biliary tract obstruction, DILI: drug induced liver injury, ERCP: endoscopic retrograde cholangiopancreatography, EUS: endoscopic ultrasound, GGT: gamma-glutamyl transferase, IIF: indirect immune fluorescence, MRCP: magnetic resonance cholangiopancreatography, PSC: primary sclerosing cholangitis, SPIU: solid phase immunoassay, US: ultrasound, +/−: positive or negative.

If the clinical scenario is suspicious for cholestasis the front-line biochemistry and serological tests are performed. First abdominal ultrasound, later magnetic resonance cholangiopancreatography (MRCP) or endoscopic ultrasound can be effective methods for imaging the intra- and extrahepatic bile ducts and serving the differential diagnostics of bile duct disorders. If the results of the serological tests and the extended imaging cannot identify the cause of the cholestasis or if autoimmune hepatitis (AIH) or nonalcoholic steatohepatitis (NASH) is also suspected liver biopsy is recommended and in rare and special cases genetic tests of monogenic cholestatic disorders can be applied. If the signs and symptoms are typical for PBC but the biochemical or available serological markers are negative, furthermore the imaging technics do not identify other liver disorders histological evaluation of liver biopsy sample can decide. When both the biochemical and serological tests are negative individual decision is required concerning liver biopsy (Fig. 1) [6,10,[29], [30], [31]]. Specific autoantibody positivity – without elevated cholestasis markers and histological alterations – is not enough alone for the diagnosis of PBC, in such cases annual control of the serum autoantibody levels and biochemistry tests for cholestasis are recommended [6,8,10,[29], [30], [31]].

Several other biochemistry markers can support the diagnostics of PBC, though they are not involved in the diagnostic criteria. Beside ALP, another marker of cholestasis, gamma-glutamyl transferase (GGT) can be elevated sometimes even prior to the rise of ALP. Cholestasis-associated parenchyma inflammation and necrosis can cause elevation of transaminases (AST: aspartate aminotransferase, ALT: alanine aminotransferase) and hyperbilirubinemia occurs as PBC progresses. Elevated international normalized ratio (INR), declining platelet count (PTC) and albumin are already signs of ongoing cirrhosis. PBC is frequently associated with elevation of serum total IgM concentration (Supplementary Table 1) [6,10,30,31].

Guidelines of AASLD and APASL mention anti-HK1 and anti-KLHL12 as promising diagnostic markers. Furthermore, cumulating data presented in section 2.3 supports that anti-HK1 and anti-KLHL12 are proper markers for helping the diagnostics of PBC in AMA-negative/ANA-negative cases, therefore we incorporated them into the diagnostic algorithm presented on Fig. 1. Since the 90-95% of the PBC cases are AMA-positive introduction of the additional autoantibodies into the diagnostic algorithm is suggested in a stepwise manner. Based on a recent study anti-gp210 and anti-sp100 together can identify 75% of the AMA-negative PBC patients while this goes up to 87.5% if we include anti-HK1 and anti-KLHL12, too [32]. This means that involvement of the PBC-specific ANAs, can enhance the sensitivity of PBC diagnostics by 3.8-7.5%, while anti-HK1/anti-KLHL12 can add an extra 0.63-1.25% to that. Since anti-gp210 and anti-sp100 can have prognostic value, too, their combined measurement with AMA – e.g. by immunoblot – suggested to be in the first line of the diagnostics, while rest of the autoantibodies should be measured only in second phase, in the AMA/ANA-negative cases. Concerning resource limited environments, this can be an even more gradual process: AMA testing followed by ANA identification and then measuring the novel autoantibodies. Though at that moment there are no studies available that compare the cost-effectiveness of AMA testing vs extended autoantibody panels or biopsy vs serological analysis, but the evaluation of ANA and the novel autoantibodies can identify up to 7.5% and 1.25% of PBC cases, respectively, where liver biopsy/histology can be spared saving money and reducing potential clinical risks. Unfortunately, at that moment only Research Use Only (RUO)-marked tests are available for anti-HK1 and anti-KLHL12 at the market, therefore, the limited availability of these tests hampers their widespread application.

## 3 PBC specific antibodies in non-PBC disorders

### 3.1 Anti-mitochondrial autoantibodies in non-PBC disorders

AMA can occasionally be detected in non-PBC subjects with other liver disease including AIH, chronic hepatitis C, chronic bacterial infection, acute liver failure, drug‐induced liver injury (DILI) and with non‐liver diseases including systemic (cutan) lupus erythematosus (SLE), Sjögren's syndrome, limited scleroderma, lymphoma and epilepsy or even in healthy people [[33], [34], [35]]. In both liver-related and systemic autoimmune diseases, incidental AMA must be interpreted with caution, as its predictive value is limited in the absence of cholestatic enzyme elevation [36].

### 3.2 Anti-nuclear autoantibodies in non-PBC disorders

The reports by Lucena and Papamichalis highlight that MND/sp100/PML-specific antibodies were detected not only in hepatic diseases (viral hepatitis, NASH), mainly PBC, but also in other clinical conditions (SLE, connective tissue diseases, skeletal diseases, lung diseases, hematological disorders) [37,38]. While anti-MND antibodies can occur in rheumatologic diseases (SLE, Sjögren's syndrome - pSS, rheumatoid arthritis - RA, mixed connective tissue disease - MCTD, vasculitis) anti**-**sp100 remains a highly specific marker for PBC and is seldom found outside hepatobiliary autoimmune diseases. Anti-MND without anti-sp100 is relatively common in systemic autoimmune disorders but reflects non-PBC antigenic targets [17,39].

Concerning ACA, they have been described in patients with a variety of rheumatologic disorders including limited systemic sclerosis, primary Raynaud's disease and pSS [40]. The study by Florin et al. demonstrated that PBC-related autoantibodies are detected in 13% of systemic sclerosis (SSc) patients, with AMA and anti-sp100 being the most frequent. Their presence showed a significant association with anti-CENP-B, but not with cholestatic liver enzyme abnormalities over three years of observation [41]. PBC-specific autoantibodies **-** particularly AMA, ACA, anti-sp100, and anti-gp210 **-** identify a distinctive subset of SSc patients characterized by milder vascular and cardiopulmonary involvement, despite the coexistence of an autoimmune cholestatic liver disease. Their longitudinal data demonstrate that both SSc**-**related and PBC-related outcomes are generally favorable in this overlap population [42].

### 3.3 Novel autoantibodies in non-PBC disorders

The work of Norman et al. provides strong evidence that anti-KLHL12 and anti-HK1 antibodies represent highly specific PBC autoantibodies. These antibodies may appear infrequently in other autoimmune or hepatic disorders (scleroderma, primary sclerosing cholangitis - PSC, acute liver failure - ALF) but are often accompanied by AMA or other PBC-related serology, suggesting potential overlap or unrecognized PBC rather than true cross-disease prevalence [24]. It was demonstrated that anti-HK1 and anti-KLHL12 can be positive in Sjögren's syndrome highlighting that anti-HK1 and anti-KLHL12 also might play a specific role in this disorder [32,43]. Anti-KLHL12, can appear in rare instances in Multisystem Inflammatory Syndrome in Children (MIS-C), though usually at low prevalence that is attributable to passive transfer via IVIG, underscoring the importance of timing and clinical context in serological interpretation [44].

## 4 Autoantibodies as prognostic and predictive markers in PBC

Risk stratification of PBC patients can be done based on special parameters that are measured dominantly at presentation or sometimes at later timepoints and their elevated/reduced value can be associated with better/worse outcome, earlier/later development of liver or extrahepatic complications, liver transplantation or death. Predictive markers forecast whether the PBC patient will react upon a certain therapy or not [10].

### 4.1 Anti-mitochondrial autoantibodies as prognostic markers

Concerning autoantibodies, the role of AMA in prognostics of PBC is rather confusing. Some studies show no association, while Chang published data showing that in an univariate analysis baseline AMA titer was associated with liver cirrhosis development [45]. More studies could show that serum AMA concentration has a correlation with biochemical prognostic markers and histological progress [46,47].

### 4.2 Anti-nuclear autoantibodies as prognostic markers

Though the number of studies is relatively low, but they suggest that ACA positivity at onset might be associated with poorer prognosis and has a significant correlation with the development of portal hypertension [22,48,49]. Furthermore, ACA can be present in 9-30% of PBC patients and in 80% of patients with PBC/SSC identifying a special overlap disorder, that can have altered clinical outcome [21,22,42,50,51]. Earlier studies showed association between ACA-positivity and development of kidney failure [52] or hepatic decompensation [53,54] in PBC patients.

Several lines of evidence support that anti-gp210 has a clear prognostic value. Haldar et al. showed that if anti-gp210 positivity was present at diagnosis the response rate to UDCA was only 16.7% compared to anti-gp210 negative patients (39.3%) [55]. The efficacy of second line therapies (OCA, fibrates, steroids) was also lower in the presence of anti-gp210 antibodies: higher levels of biochemical markers and increasing rates of complications, transplantation and mortality was observed [56,57]. In other studies and in a meta-analysis of 5 publications anti-gp210 positivity was also associated with poor prognosis, a high risk of liver-related death and transplantation [19,[58], [59], [60], [61], [62]]. Nakamura et al. even proposed two types of disease progression, a hepatic failure type in the presence of anti-gp210 antibodies and a portal hypertension type associated with ACA [22], that was supported by a recent study [49]. Bauer and colleagues showed that anti-gp210 alone did not associate with prognosis of PBC while the combination of 2 or 3 autoantibodies against the nuclear pore complex (anti-gp210, anti-LBR, anti-p62) resulted in a shorter survival time and shorter time for liver transplantation [63]. Wang and colleagues showed, that the longitudinal presence of anti-gp210 antibodies in UDCA treated patients associated with a higher risk for development of end stage liver disease [62]. Few other studies had contrasting results to those mentioned above [23,64,65].

Anti-sp100 positivity has also some prognostic value in PBC patients. Reig and colleagues showed that transplant-free survival and time to liver decompensation was significantly lower in patients with high anti-sp100 positivity, and the rate of patients with a favorable GLOBE-PBC score was significantly lower in this group [23]. Bauer and coworkers could show that any of the anti-PML-NB-specific antibodies has no prognostic value on its own, but the presence of two or three antibody positivity was associated with higher rate of death and need for transplantation, in this way with a more progressive liver disease [66]. Similarly, Züchner et al. could show that the presence of anti-sp100 and anti-PML antibodies was associated with a worse disease course since unfavorable histological changes were observed in this group more frequently [67]. Another study had contrasting results to those mentioned above [65].

### 4.3 Novel autoantibodies as prognostic markers

Anti-HK1 positivity can also have some prognostic value. It is described to be associated with a higher frequency of liver decompensation and a lower rate of transplantation free survival [23]. Contradictory data were published for anti-HK1 in another study and in this report anti-KLHL12 showed no association with risk assessment scores, too [32].

### 4.4 Current guidelines about the prognostics of PBC

According to the guidelines of EASL, AASLD and APASL the classical bad prognostic markers in PBC are male sex, younger age, smoking, alcohol consumption, early onset of symptoms, fatigue and pruritus at onset and advanced liver damage stage at diagnosis. Several qualitative binary scores and continuous scoring systems containing biochemical parameters (bilirubin, ALP, GGT, AST, albumin, PTC tests) are available - calculated pre- or post-therapy - that can identify individuals with worse prognosis. Serum bilirubin concentration is the best prognostic marker for survival, though its concentration is elevating only at later stages of the disease, while elevated ALP level is the best predictive marker for treatment response [6,10,30,68]. Cirrhosis and portal hypertension are associated with elevated bilirubin, immunoglobulins, hyaluronic acid, INR and with reduced albumin and thrombocyte count. Fibrotic remodeling of the liver can be evaluated by the Enhanced Liver Fibrosis (ELF) test, by the Aspartate Aminotransferase-to-Platelet Ratio Index (APRI), or by liver stiffness measurement (LSM) assessed by vibration-controlled transient elastography (VCTE). High baseline values of these parameters are also associated with worse prognosis [6,10,30,68]. Finally, advanced PBC-specific histology stage at onset is also a bad prognostic marker, though liver biopsy is taken less frequently nowadays (Supplementary Table 1) [6,10,30,68].

**Fig. 2:**
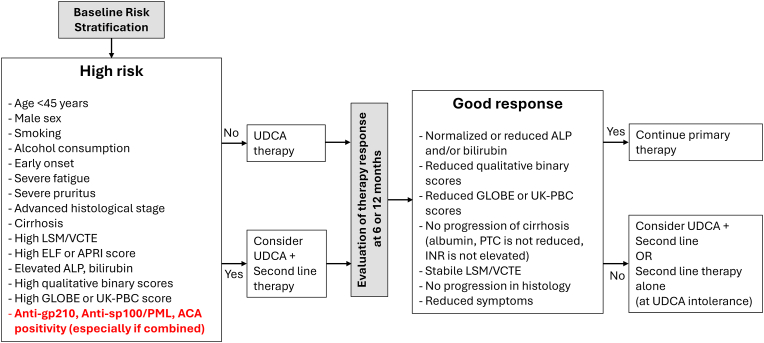
Prognostics and follow-up of patients with PBC. Decision about first line therapy is determined by prognostic factors at the onset of disorder. These include – in the order of their importance – i) biochemical markers and their combinations, ii) state of liver fibrosis/cirrhosis determined by biochemical tests, elastography and histology iii) severity of clinical symptoms. Certain serological parameters (anti-gp210, anti-sp100, ACA) are suggested to be involved in this decision in the fourth line. Lack of progression in these parameters – except autoantibodies – measured at 6 or 12 months can serve the evaluation of therapy efficacy and can support second line therapy selection. ACA: anti-centromere antibody, ALP: alkaline phosphatase, anti-PML: anti-promyelocytic leukemia protein, APRI: aspartate aminotransferase to platelet ratio index, ELF: enhanced liver fibrosis, GGT: gamma-glutamyl transferase, INR: international normalized ratio, LSM: liver stiffness measurement, PTC: platelet count, UDCA: ursodeoxycholic acid, VCTE: vibration-controlled transient elastography.

Concerning clinical decision making about the therapy to be chosen biochemical markers (alkaline phosphatase, bilirubin, albumin) and the qualitative and quantitative scores derived from them are the most valuable parameters. The fibrotic/cirrhotic stage of the liver evaluated by histology (if liver biopsy sample is available) or elastography or by the ELF and the APRI tests are also highly informative. As a third line, the clinical scenario (age, gender, symptom profile) and additional histological alterations also have to be taken into account. All three international guidelines and cumulating data presented in section 4.2 support that anti-gp210, anti-sp100/PML and ACA positivity can be involved in prognostic evaluation of contradictory cases though with much lower power compared to the classical markers (Fig. 2) [6,10,30,68].

## 5 Evaluation of disease activity

### 5.1 Anti-mitochondrial autoantibodies as activity markers

Monitoring disease activity by AMA is not part of the clinical practice [30,31,68], though there are some reports describing some correlation with AMA concentration and disease activity and/or its biochemical and histological markers. Alfano and colleagues noticed higher AMA-M2 values in sera of patients with progressive disease state or uncontrolled by therapy [69], Gabeta and colleagues showed that the presence of AMA IgG is associated with more severe disease [70], Gatselis et al. described that the changes of AMA titer was associated with disease severity [71], Wang et al. could show that the degree of cholestasis and hepatic injury is associated with the AMA levels [72], while Dellavance and colleagues found higher AMA levels in patients with abnormal biochemistry [73].

### 5.2 Anti-nuclear autoantibodies as activity markers

Several lines of evidence indicate that anti-gp210 is the most valuable autoantibody, showing clear association with disease activity/severity. Biochemical markers (ALP, bilirubin, GGT, AST, ALT, IgM, IgG) were significantly higher in anti-gp210 positive PBC patients in several studies [19,20,55,59,62,72,74,75]. Nakamura and colleagues could also prove that the anti-gp210 positivity was associated with more severe histological alterations in the liver [75,76].

Bauer et al. studied autoantibodies against the three members of the PML Nuclear body complex: anti-sp100, anti-PML, anti-sp-140. They could show that the presence of any of these antibodies is associated with higher serum bilirubin and ALP, and more severe histological grade [66]. Similarly in an early study Mytilinaiou et al. could show that anti-sp100 levels correlated with the Mayo risk score and combined anti-PML+/sp100+ patients had more advanced disease compared to patients negative for these antibodies [77].

ACA did not show association with disease activity markers in earlier studies [21,28,78].

### 5.3 Novel autoantibodies as activity markers

Significant correlations could be observed between anti-HK1 antibody expression and the serum levels of AST, ALP, GGT and bilirubin suggesting that the presence of this antibody can correlate with disease severity [79]. Data about anti-KLH12 antibodies is also sparce. Patients with these antibodies had increased liver fibrosis with advanced histological stages (III/IV) and elevated levels of serum bilirubin, AST, ALP and GGT [63,79].

### 5.4 Current guidelines about the activity markers of PBC

The guidelines of the 3 international societies (EASL, AASLD and APASL) do not discuss separately disease activity and disease follow-up markers but evaluate them in the context of therapy efficacy monitoring. The classical markers of disease activity in PBC are biochemical parameters: i) ALP, GGT, bilirubin are markers of cholestasis, ii) ALT and ALP elevated upon hepatocyte damage, iii) elevated IgM associated with inflammation and immune activation, iv) while reduced albumin, platelet count and elevated prothrombin time (INR) are already signs of liver cirrhosis. Several qualitative binary scores and continuous scoring systems are created from these parameters showing association with disease activity. Some US-based diagnostic techniques (LSM/VCTE) are available for evaluation of the rate of liver fibrosis, too. More precise evaluation of disease activity can be done by histopathological evaluation of liver biopsy samples, but this requires invasive sampling associated with unneglectable risk [6,10,30,68,[80], [81], [82]].

Though anti-gp210, anti-sp100 and even AMA could show association with biochemical or histological markers of disease activity unfortunately there are not enough conclusive, longitudinal studies where these autoantibodies were measured at several time-points and could judge reliably therapy efficacy, as we show it in Section 6.

## 6 Follow-up, evaluation of disease progression and therapy response

### 6.1 Therapeutical approaches in PBC

The outcome in the case of untreated PBC patients is heterogeneous but clearly poor. The average survival time in these individuals is 9-16 years, proper and early therapy and life-long patient care are essential. The major aim of the treatment of PBC is to prevent or at least slow down the progression of the liver disease and relieve the disease associated complaint that reduce the patients' quality of life. The only “curative” therapy at that moment is liver transplantation, though the 5-year survival is 80-85% and the PBC relapse rate, after 15-years is up to 40% [10].

The first line therapy for PBC is ursodeoxycholic acid (UDCA), that has a choleretic effect and protects hepatic epithelial cells from toxic effect of natural bile acids. UDCA can effectively slow down disease progression and can delay liver transplantation. Several biochemical markers can be reduced by UDCA therapy, and these can be used to monitor efficacy of treatment. These dominantly include ALP, GGT, bilirubin and albumin and at least seven different UDCA response criteria have been defined so far. The efficacy of therapy has to be evaluated at 6 and/or 12 months after its initiation. The values of these markers can predict also the potential further efficacy of UDCA [83,84]. Recently, two continuous scoring systems were developed. The GLOBE (age at diagnosis, ALP, bilirubin, albumin and platelet count at 12 months) and the UK-PBC (the baseline albumin and platelet count plus ALP, bilirubin and AST (or ALT) at 12 month) risk score systems can suggest for the physician that the response to UDCA will be incomplete and it is feasible to change to a combination therapy [[85], [86], [87], [88]].

Until 2024/2025 the most frequently used second-line therapy was obeticholic acid (OCA), that is a semi-synthetic hydrophobic bile acid analogue. It is highly selective for the farnesoid X receptor (FXR) involved in inflammatory reactions and metabolic regulation of hepatocytes. Furthermore, it can efficiently alter the biosynthesis of natural bile acids. Unfortunately, OCA caused pruritus, resulting in termination of therapy in 10% of the cases, furthermore patients with decompensated cirrhosis receiving OCA could develop severe liver injury. After termination of a recent confirmatory trial OCA was withdrawn and it is not available for treatment of PBC patients [89]. A third option is to combine UDCA therapy with fibrates, targeting peroxisome proliferator activated receptors (PPARs), which affect bile acid synthesis and detoxification. According to current suggestions PBC patients with an inadequate biochemical response or intolerance to first-line UDCA therapy should be considered for second-line treatment with PPAR agonist, including elafibranor or seladelpar (both conditionally approved) or bezafibrate (off-label use). Lastly, budesonide, a second-generation corticosteroid can be combined with UDCA. This drug has less systemic side effects and more liver-specific actions but cannot be used in cirrhotic patients. The biochemical markers, and risk scoring systems of therapy efficacy are similar to those used for UDCA [6,90].

### 6.2 Anti-mitochondrial autoantibodies in the follow-up

Concerning AMA in separate studies Poupon, Tang, Kisand and Wang showed that AMA levels were decreased upon UDCA treatment in association with the biochemical markers [72,[91], [92], [93]], while in 6 other studies this association was not observed [64,[94], [95], [96], [97], [98]]. Higher AMA titers were associated with advanced disease state during follow-up [71]. In a recent, large, cohort study of 28 years Chang et al. showed that after 1 year of UDCA treatment only UDCA responders had a decreased AMA titer [45]. Rituximab therapy reduced the number of B cells producing AMA and serum AMA levels showing association with histological alterations [99], while upon combined UDCA + fenofibrate therapy half of the asymptomatic patients had decreased AMA titer [100].

### 6.3 Anti-nuclear autoantibodies in the follow-up

Concerning anti-gp210 Wang and colleagues showed that the level of this antibody fluctuated during UDCA therapy showing association with response to therapy [62,72]. Similar results were provided by Nakamura et al. and Wesierska-Gradek et al. [22,56,75,101].

Anti-sp100 antibody titers also showed association with UDCA efficacy. An increase in its serum concentration was associated with a poorer outcome in one study [23], while a decrease in its titer was linked to a better response to UDCA therapy [102]. In another study of Gatselis et al., decrease in anti-sp100 level was associated with a reduced Mayo score and a better response to UDCA [71]. In a recent study Wang and colleagues could show that after at least 6 months of UDCA treatment anti-sp100 level was significantly lower than at initiation of therapy [72]. In contrast, Zuchner et al. and Mytilinaou et al. in two earlier studies showed that the level of anti-sp100 is stable during follow-up [67,77], while Tana and colleagues presented that anti-sp100 decline was associated with enhanced liver fibrosis and cirrhosis [64].

### 6.4 Other autoantibodies in the follow-up

Anti-HK1 positivity was associated with a lower response to UDCA treatment [23]. Anti-KLH12 levels were rather stable during the follow-up and did not show association with disease progression and outcome [63].

### 6.5 Current guidelines about the follow-up of PBC patients

According to the guidelines of EASL, AASLD and APASL the efficacy of therapy is most frequently monitored by biochemical parameters at 6 or 12 months after initiation of the treatment. Adequate response can be defined if the ALP concentration is < 1.5-3 x ULN (upper limit of normal) or reduced by 15-40% compared to the base-line value or if serum bilirubin concentration is < 1 x ULN, depending on the applied response criteria systems [30,90]. Frequently AST and ALT are reduced significantly upon UDCA or OCA therapy. All these biochemical changes are associated with improved hepatic outcome and reduced clinical events [74]. Furthermore, the combination of these markers as qualitative binary scores or continuous scoring systems can support clinical decisions. If no progression of liver cirrhosis (albumin, PTC is not reduced, INR is not elevated) and fibrosis (stabile LSM/VCTE) is observed that means also a good response. Though biopsy is rarely taken during follow-up, but no progression in histology also proves the efficacy of therapy (Supplementary Table 1) [6,10,30,69].

None of the 3 guidelines mentioned autoantibodies among biomarkers that can be used for follow-up of PBC patients [6,10,30,69]. In contrast there are studies – mentioned in section 5.2 and section 6.3 – showing that anti-gp210 were associated with other markers of disease activity even in follow-up studies, too. In spite of these the cumulated evidence is still not enough to suggest reduced levels of anti-gp210 or its negativity as a response marker in PBC. Nonetheless, anti-gp210 is the most promising marker to be involved in the follow-up of PBC therapy, but additional studies are required to support this notion. Unfortunately, hardly any ongoing trials aim the evaluation of the efficacy of autoantibodies in therapy response. At the clinical trial webpage of NIH National Library of Medicine (https://clinicaltrials.gov) the search “Primary biliary cholangitis/PBC” AND “antibody” provides 29 trials, from which only three (NCT00490620, NCT01614405, NCT02376335) follow AMA longitudinally during evaluation of a new therapeutical approach. This is not surprising since identification of soft outcome measures in PBC therapy response evaluation is not a real unmet clinical need, as biochemistry markers – especially their combined qualitative and quantitative scores –, and the US based parameters of liver fibrosis (LSM/VCTE) function quite well for treatment follow-up. Furthermore, recruiting patients for a second-line PBC therapeutic trial is problematic knowing the low number of PBC patients and the high rate of good response to UDCA treatment.

## 7 Monitoring complications

### 7.1 Hepatocellular cancer (HCC)

The incidence of HCC among PBC patients is between 2.4 and 6.6 cases per 1000 individuals, that is somewhat lower than in the case of other liver disorders but higher than in the average population. The risk factors for HCC development are male sex, advanced histological stage, UDCA treatment failure, older age, portal hypertension, HBV infection, diabetes, obesity and alcohol consumption [6]. Concerning autoantibody positivity as prognostic marker for HCC data are confusing. Reig et al. could not find relation between development of HCC and antibody seropositivity [23], while Chang and colleagues showed a positive association of AMA titer and HCC development in an univariate analysis [45]. One study showed that the frequency of ACA positivity is higher in PBC patients without than with HCC [103]. Based on these, regular screening for HCC by abdominal imaging is suggested by every 6 months for males and patients with cirrhosis. This can be complemented by serum alfa-fetoprotein measurement, but autoantibodies are not included in the current guidelines [31].

Beside HCC also solid tumors might develop in PBC patients, that has a higher risk compared to the general population (relative risk: 1.55) [104]. In a recent study of Bian et al., from 580 PBC patients 46 had solid tumors (colorectal, breast, gastric, HCC, papillary thyroid, cervical, lung and kidney cancer) and in 29 patients tumor developed after diagnosis of PBC. In the tumor negative cases, ACA positivity was more frequent and was a protective factor [103]. Taken together during follow-up of PBC patients not only HCC but solid tumors should be considered.

### 7.2 Recurrence of primary biliary cholangitis after liver transplantation

Liver transplantation (LT) can be the ultimate therapy of PBC patients with decompensated liver cirrhosis or complications secondary to portal hypertension (MELD score >15, Mayo risk score >7.8, bilirubin >6 mg/dL, unbearable pruritus). HCC is an exceptionally rare indication for LT in PBC patients. The 5-year survival rate is about 80-85% in these patients, but the recurrence rate of PBC is clearly increasing in time: 22% after 5 years, 21–37% after 10 years and 40% after 15 years. The median time for recurrence is 3 to 6 years [30,68,105]. The risk of recurrence is associated with tacrolimus therapy, MELD score and older donor age [30,68]. Recurrence can be noticed in the elevation of the biochemical markers but not in all cases and can be based on histological evaluation, since biochemical markers can rise due to several other reasons (rejection, GVH disease, DILI, biliary obstruction) [105]. AMA and PBC specific ANA can remain elevated or reappear – without the presence of PBC – after liver transplantation, in this way cannot be used for diagnostic purposes. Serum IgM elevation can be observed in recurrent PBC and therefore it is a good indication of liver biopsy [6,30,68,90].

## 8 Monitoring risk populations for PBC

### 8.1 AMA-negative PBC

The rate of AMA-negative PBC is about 5-10%. Some of these cases can be associated with other PBC-specific antibody positivity – even with higher levels –, but still there are individuals who are negative for all of the widely tested autoantibodies [106].

Concerning the outcome in a study of 521 dominantly male PBC patients the AMA-positive and AMA-negative groups (ANA test results are unknown) did not show significant differences in the occurrence of liver-disease related death, HCC or decompensation and had a similar rate of UDCA response [107]. Similar data were presented by Liu and colleagues, showing no significant difference in clinical manifestations, liver biochemistry and histological findings between AMA-negative and AMA-positive PBC patients. Furthermore, the response rate to UDCA treatment was similar in the 2 groups after a 1-year-long follow-up [108]. Cançado et al. supported these observations showing slight differences in some biochemical markers but similar rate of liver-related complications, liver transplantation and death [109]. In one of the most recent studies Liu et al. showed that AMA- and ANA-negative PBC patients had slightly lighter pathological changes observed in liver biopsy samples compared to the autoantibody positive individuals. Concerning the biochemical parameters and the clinical features only the serum IgM level was significantly lower in the AMA/ANA-negative patients [110]. No significant difference was observed between AMA-positive and AMA-negative patients in biochemical, histological and clinical features in two other reports [111,112]. In contrast to all these studies, Juliusson et al. evaluated 71 AMA-negative and 71 AMA-positive PBC patients and the former ones had a significantly reduced survival, free of liver related complications, showing a worse prognosis [113], while Jin and colleagues could show a significantly lower bile duct damage around the portal areas in AMA-positive PBC than those observed in AMA-negative patients [114].

In summary, the majority of the studies show that the clinical course and histological features of AMA-negative and -positive PBC do not differ significantly and the response to UDCA treatment seems to be similar in both groups. Anyway, autoantibody-negative ALP/GGT liver enzyme elevations associated with clinical symptoms of PBC require liver biopsy for early diagnosis [6].

### 8.2 AMA-positive cases without PBC

Several lines of evidence indicate that individuals with AMA-positivity but without ALP/GGT elevation do not show clinical features of PBC and only small percentages of these patients will progress to PBC, based on biochemical tests [6,30]. In a French nationwide cohort of 1,318 AMA-positive individuals identified by indirect immunofluorescence (titer ≥1:40), only 16% of those with normal alkaline phosphatase levels developed PBC within five years, while the majority of them remained clinically stable [115]. Similarly, a six-year Austrian follow-up study of 184 AMA-positive individuals revealed that only 10% developed new-onset PBC while 9% reverted to AMA negativity. This data also suggest that AMA may sometimes be a transient, nonpathological finding [116]. A Chinese cohort of 139 AMA- or AMA-M2-positive patients showed a 4.3% progression rate to PBC after a median of 4.6 years. Elevated IgM and low ALT levels were independent predictors of progression; none of the patients with coexisting non-PBC liver diseases developed PBC [36]. In contrast, a Turkish study by Ergenc et al. reported a higher short-term risk among 48 AMA-M2-positive individuals identified by immunoblot. Thirty-one percent of these individuals developed definite PBC within 27 months. ANA positivity and other autoimmune comorbidities were common in this group [117].

On the other hand, much higher frequency of PBC diagnosis was established, when liver biopsy was also taken from the AMA-positive and/or ANA-positive but ALP-negative individuals. A recent study of 165 such patients concluded that more than half of them (53.3%) had biopsy-confirmed PBC. AMA-M2 positivity emerged as a key indicator of underlying disease, particularly when accompanied by the presence of anti-gp210 or anti-sp100. These patients had the highest likelihood of PBC (82.1%), while isolated AMA-M2 positivity still carried a substantial risk (48.4%). Elevated IgM (>0.796 × ULN) and the absence of alternative liver disease etiologies were independent predictors of PBC [118]. Similar data were presented by some earlier reports. Mitchinson et al. showed a 82.8% diagnosis rate in 29 AMA+/ALP- patients [119]*,* while Sun and colleagues a 82.1% rate (55 form 67 similar patients) [120]. The study by Berdichevski et al. presented six AMA-positive patients with normal ALP, and four of them (67%) had florid bile duct lesions compatible with early PBC. Those with biopsy-proven PBC had higher AMA titers and serum IgM levels than biopsy-negative subjects [121].

Taken together, the literature provides conflicting evidence regarding the likelihood of PBC development in AMA-positive patients without biochemical evidence of cholestasis, with reported progression rates ranging from 4% to 31% based on biochemistry markers, while 50% to 80% based on liver biopsy [36,[115], [116], [117]]. Moreover, the underlying risk is strongly influenced by the autoantibody profile. In particular, AMA-M2 positivity, especially when combined with anti-gp210 or anti-sp100, substantially increases the likelihood of underlying, biopsy-proven PBC, even in individuals with normal ALP levels [[118], [119], [120], [121]]. Despite the considerable variability in published progression rates, patients with AMA-M2 positivity, multiple autoantibody positivity, high antibody titers or elevated IgM levels appear to be at higher risk. These patients should undergo annual biochemical assessment and liver biopsy when clinically indicated [6,30].

### 8.3 Family members of PBC patients

Large epidemiological studies consistently demonstrate marked familial aggregation of PBC. In a European case–control study, a family history of PBC was associated with an odds ratio (OR) of 6.8 for PBC, and a family history of autoimmune thyroid disease further increased susceptibility (OR 7.1) [122]. In a large, controlled interview-based study, a self-reported history of PBC in a first-degree relative was associated with a more than tenfold increased disease risk (adjusted OR 10.7, 95% CI 4.2–27.3) [123] and similar data were provided by a Swedish study [124]. Familial clustering is not limited to first-degree relatives. Using a nationwide Icelandic genealogical database, the relative risk of PBC was 9.1 among first-degree relatives, 3.6 among second-degree relatives, and 2.6 among third-degree relatives, indicating a graded decline in risk with increasing genetic distance [125]. Consistent with these epidemiological observations, genome-wide association studies have identified multiple HLA and non-HLA loci associated with PBC risk [6].

In addition to clinically overt disease, subclinical autoimmunity is frequently observed among family members. Antimitochondrial antibodies are detected in approximately 13% of first-degree relatives, compared with about 1% of matched controls. The highest prevalence of AMA is observed among female relatives, particularly sisters (20.7%), mothers (15.1%), and daughters (9.8%), with lower frequencies in male relatives (brothers 7.8%, fathers 3.7%, sons 0%) [126]. In a cohort of Greek first-degree relatives, AMA-positive individuals usually had normal or only mildly abnormal liver biochemistry, while PBC-specific ANAs were largely absent, supporting the concept of a preclinical stage of familial susceptibility [127].

Longitudinal follow-up studies provide important insight into disease progression among at-risk relatives. Over a median follow-up of approximately nine years, the cumulative incidence of clinically manifest PBC among first-degree relatives remained low. Progression to overt disease occurred in approximately 4% of seropositive relatives, while no cases were observed in those with normal baseline alkaline phosphatase levels, indicating that isolated AMA positivity does not reliably predict short-term disease progression [128].

Further evidence for a strong genetic contribution is provided by studies in twin cohorts, demonstrating a high concordance rate for PBC among monozygotic twins (63%) and no concordant cases among dizygotic twins, indicating a major genetic contribution modulated by epigenetic and environmental factors [129].

In summary, evidence from epidemiological studies, population-based registries, serological analyses, longitudinal follow-up, and twin studies indicate that family members of patients with PBC represent a well-defined high-risk group characterized by prolonged preclinical autoimmunity and heterogeneous disease progression. These observations support targeted surveillance strategies in selected high-risk individuals, consistent with guideline recommendations [30,130,131]. In particular, female first-degree relatives over the age of 30 should be screened for ALP and, upon its elevation, for AMA regularly. If AMA is negative, these tests should be repeated in 5-year intervals [30].

## 9 Why do the results of autoantibody testing provide contrary data in PBC studies?

**Fig. 3:**
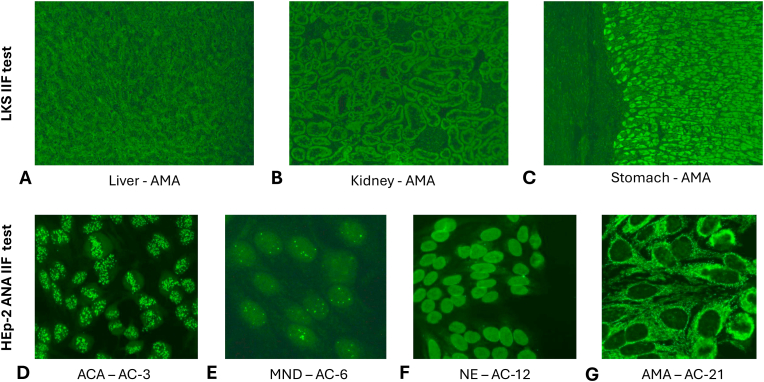
Microscopic patterns observed in the presence of PBC-specific antibodies using rodent LKS (A,B,C) and HEp-2 ANA (D,E,F,G) indirect immunofluorescence methods. Anti-mitochondrial antibodies (AMA) show granular staining in the cytoplasm of hepatocytes (liver, A), tubular epithelial cells (kidney, B) and parietal and chief cells (stomach, C). A coarse granular filamentous staining is observed in the cytoplasm of HEp-2 cells (G). PBC-specific ANA-patterns are multiple nuclear dots (MND, AC-6, E) and punctate nuclear envelope (NE, AC-12, F). Anti-centromere antibodies (ACA, AC-3, D) can occur frequently in PBC, too.

Though data are accumulating about the different applications of autoantibody testing in PBC, drawing a solid conclusion is impeded by the large variation observed in these studies. One explanation can be a methodological problem. PBC-specific autoantibodies can be determined by different immunoassays. Indirect immunofluorescence (IIF) on rodent liver, kidney and stomach tissues is considered as a screening test, having a relatively lower analytical sensitivity but higher diagnostic specificity and can identify different types of AMAs (Fig. 3.A,B,C) [8,29,132]. PBC-specific ANA-s can be detected by the rodent triple-tissue IIF test, but currently their identification is based on IIF assays utilizing HEp-2 cells. These latter tests have a higher sensitivity for these antibodies (AC-6 and AC-12) and higher titers of AMA can be also noticed (Fig. 3. D, E, F, G) [8,29]. The solid phase immunoassays (ELISA, ECLIA, CLIA) can quantitate the level of these autoantibodies separately in individual single assays but multiplex systems are also available providing concentration of several different autoantibodies in one run. These assays have usually a much higher analytical sensitivity compared to IIF tests, but this can enhance the rate of false positive test results, too [8,29,70,133]. Line- or dot-immunoblots can identify several autoantibodies at a time, but provide data only on a semiquantitative scale, with a middle level sensitivity. The differences in the sensitivity and specificity of the different types of methods used for antibody determination is an important factor explaining heterogeneity among the different studies [8,29,134]. Furthermore, even a certain type of assay (e.g. ELISAs) provided by different manufacturers can give scattering test results even in the same patient cohort, emphasizing the need for standardization of these autoantibody detecting assays [29,135,136]. The most important properties of methods used for determination of PBC-specific autoantibodies are presented in Table 2.

**Table 2:** Essential features of the methods used for detection of PBC-specific autoantibodies.

Method	Indirect immunofluorescence test on HEp-2 cells	IIF - LKS tissues	Immunoblot/line immunoassay	Enzyme/luminescence/fluorescence immunoassay (ELISA, LIA, FEIA)
**Antibody**	- nuclear body - multiple nuclear dots (AC-6): anti-sp100, anti-PML, anti-sp140 - nuclear pore complex - punctate nuclear envelope (AC-12): anti-gp210, anti-p62, anti-LBR - kinetochore - centromere (AC-3): anti-CENP-A/CENP-B - mitochondrion - cytoplasmic reticular (AC-21): AMA	- AMA	- AMA-M2 - anti-sp100 - anti-PML - anti-gp210 - anti-HK1	- AMA-M2 - anti-gp210 - anti-sp100 - anti-KLHL-12 - anti-HK1
**Analytical sensitivity**	low	low	middle	high
**Analytical specificity**	low	low	middle	middle
**Antigen source**	human epithelial cell layer	rat/mouse tissue	purified animal or recombinant human	purified animal or recombinant human
**Number of antibodies detected**	multiple	multiple	multiplex	single
**Quantitation**	qualitative or semiquantitative (titer)	qualitative or semiquantitative (titer)	qualitative or semiquantitative (negative, +, ++, +++)	quantitative (arbitrary unit)
**Standardization of method**	International Consensus on ANA Patterns (ICAP)	-	-	-
**International control sample**	IS2724 Anti-Mitochondrial (AMA) (AC-21), IS2728 Anti-MND (AC-6), IS2134 Anti-Centromere (AC-3)	-	-	-
**Which autoimmune hepatitis specific antibodies can be detected?**	- nuclear homogeneous (AC-1) - actin-like (AC-15)	- SMA - anti-LKM - anti-LC	depends on reagent	none
**Can unknown/uncharacterized antibodies be detected?**	yes	yes	no	no

AMA: anti-mitochondrial antibody; HK-1: hexokinase 1; IIF: indirect immunofluorescence; KLHL12: kelch-like 12 protein; LBR: lamin-B receptor; LC: liver cytosol; LKM: liver-kidney microsomal; LKS: rodent liver, kidney, stomach; MND: multiple nuclear dots; PML: promyelocytic leukemia proteins; SMA: smooth muscle antibody; sp100: nuclear body speckled 100 kDa.

Standardization of a laboratory test - in the metrological sense - necessitates a reference standard, which ensures comparability of results through traceability of calibrations. Theoretically it is impossible to make such a reference material in autoantibody testing because the analyte is ill-defined. The antigen target is usually a protein with multiple epitopes including conformational ones whose structure is hard to preserve if bound to a solid phase which is used in most assays. Antibodies produced by patients also differ in affinity, isotype, subclass, allotype, glycosylation etc. [137,138]. Though there are projects still targeting the development of international reference materials [[139], [140], [141]], but most organizations dealing with compatibility of autoantibody results switched their goal from standardization to harmonization, which includes finding and minimizing differences in test ordering, type of test, assay protocol, result reporting and interpretation [142]. The International Consensus on ANA patterns (ICAP) initiative could develop a clear system for describing ANA HEp-2 IIF patterns (www.anapatterns.org) that can help the result reporting in autoimmune diagnostics [143]. Several projects of the European Autoimmunity Standardisation Initiative served a similar purpose in other areas of autoimmune testing [136,142,144]. A novel approach in harmonization of results provided by solid phase immunoassays is the introduction of likelihood ratios in test reporting [145].

The other factor influencing study outcomes is the patient population. Patients with PBC are rather rare, because of this several studies in literature involved only small cohorts with a lower statistical power [6,146]. This can be a clear problem testing the second lines of therapeutical agents, since most patients react to UDCA quite well and the number of patients on other therapies is rather rare [147]. Furthermore, identification of activity markers depends on whether the disease activity was based on biochemical markers or histopathological features, and the evaluation of prognostics is altered by the chosen outcome (e.g. liver-related complications, transplantation or death) [28,146,148,149].

## 10 Future perspectives

Indirect immunofluorescence tests remain mandatory in PBC diagnostics but long assay time and need of a specialized expert in the reading phase make this method cumbersome especially in big laboratories. Automation of slide preparation and use of automated fluoresce microscopes are becoming increasingly widespread. Computer-aided pattern recognition using machine learning algorithms and artificial intelligence can significantly help in reading IIF slides [150,151]. Automated pattern identification is usually available only for cellular substrates, which means that ANA (HEp-2 cells) and ANCA (granulocytes) detection can be fully automated if PBC is suspected, however, computer generated results should be validated by an expert [144,152]. Pattern recognition in IIF on rodent liver-kidney-stomach tissue sections is usually not available, most automated microscopes only take photos of several fields of view and present the stitched panorama image on a computer screen for human evaluation. Results of studies comparing human versus computer aided or generated LKS IIF test evaluation are hardly available.

Multiplex testing has the advantage of high throughput screening for PBC on limited volume of sample detecting all relevant antibodies in one step [153]. Use of immunoblot technique for multiplexing is limited by being a qualitative or semiquantitative test, although there are reports showing low variability of blot quantification [154]. Quantitative multiplex platforms available and appropriate for PBC diagnostics use several groups of autoantigen coated beads (Luminex) or particles (Aptiva) and differentiate them based on distinct fluorescence signature [72,155]. Advantage of this approach is the possibility of testing autoantibodies to more antigens or even epitopes than which is possible with combination of single tests. Oertelt et al. found that using a bead assay to detect antibodies to the three mitochondrial autoantigens, PDC-E2, BCOADC-E2 and OGDC-E2, one fifth of the AMA-negative patients had antibodies to at least one [156]. Antibody profiling using the automated particle-based multi-analyte technology (PMAT) to determine anti-MIT3, anti-sp100, anti-gp210, anti-HK1 and anti-KLp antibodies resulted in higher recognition rate of AMA-negative PBC patients than with singleplex, conventional immunoassays [155]. Detecting all relevant autoimmune liver disease specific autoantibodies at once has the power to recognize concomitant autoimmune hepatitis. However, adjustment of cut-off values is of utmost importance to reduce the rate of false positive incidental findings. Combined evaluation of multiple biomarkers has also the potential to identify disease subtypes (cluster analysis). A recent report enrolling 537 patients used cluster analysis on 19 autoantibodies measured routinely and could identify 5 clusters of PBC. Anti-gp210, ACA, AMA-M2, anti-Ro52 were the dominant autoantibodies in the models, creating distinct PBC subgroups with special clinical scenario and different outcomes. Anti-SSA and anti-SSB were associated with PBC-Sjögren's syndrome overlap. Based on these data the combined application of different PBC-specific autoantibodies can identify individuals with a worse prognosis more efficiently [157].

## 11 Conclusions

Concerning PBC **diagnostics,** AMA and PBC-specific ANAs have a clear role in this process, and these antibodies are included in the current guidelines, too. The novel markers (anti-HK1 and anti-KLHL12) can help with the identification of AMA-negative/ANA-negative cases. The **prognostic and predictive** utility of the autoantibodies is getting more recognized in this disorder. Most evidence is accumulated about anti-gp210 as a bad prognostic and predictive marker showing a liver-failure type outcome of the disease, while ACA was associated with a portal hypertension type progression. High levels of anti-sp100 showed an association with shorter time to decompensation and liver transplantation and this was even more expressed when two or more members of the PML nuclear bodies were targeted by autoantibodies (e.g. anti-PML or anti-sp140). Concerning **disease activity** practically all the classical and novel autoantibodies could show association at least with elevated levels of the biochemical markers of disease activity or sometimes with worse histopathological features, too. Several lines of evidence indicate that elevated levels of AMA, anti-gp210 and anti-sp100 correlate also with a more advanced clinical state of PBC. The usefulness of antibodies in **follow-up** of patients during therapy is less clear due to the lower number and contradictory studies. Results are most promising in the case of anti-gp210 showing fluctuating but declining concentration during UDCA therapy in association with response to therapy. In some **special clinical scenarios** autoantibody testing can help clinicians. AMA-positive individuals without any clinical signs and biochemistry marker positivity should undergo annual biochemical assessment and liver biopsy when clinically indicated and this is even more relevant in relatives of PBC patients.

Though data are accumulating about the different applications of autoantibody testing in PBC drawing a solid conclusion is impeded by the large variation observed in these studies. This inconsistency is dominantly caused by inhomogeneity of the tested patient populations and the large difference in the results provided by the disparate methods used. Because of these, for a solid evaluation of the potential roles of autoantibody testing in the clinical management of PBC large prospective studies are required involving many well-characterized PBC patients, with clearly defined activity and outcome parameters using highly standardized, sensitive immunoassays for autoantibody determination.

## CRediT authorship contribution statement

**Péter Antal-Szalmás:** Writing – review & editing, Writing – original draft, Visualization, Supervision, Methodology, Investigation, Conceptualization. **Dóra Bencze:** Writing – original draft, Investigation. **Sarolta Demeter:** Writing – original draft, Investigation. **Krisztina Pénzes-Daku:** Writing – original draft, Investigation. **Lilla Szabó:** Writing – original draft, Investigation. **Beáta Tóth:** Writing – original draft, Investigation. **Róza Földesi:** Writing – original draft, Investigation. **Mária Papp:** Writing – review & editing, Conceptualization. **Gábor Nagy:** Writing – review & editing, Writing – original draft, Visualization, Methodology, Investigation, Conceptualization.

## Declaration of generative AI and AI-assisted technologies in the manuscript preparation process

During the preparation of this work the authors used CHATGPT in order to support literature search in the internet. After using this tool/service, the authors reviewed and edited the content as needed and take full responsibility for the content of the published article.

## Funding

The publication costs of the article are covered by the Science support program of the 10.13039/501100009232University of Debrecen.

## Declaration of competing interest

The authors declare that they have no known competing financial interests or personal relationships that could have appeared to influence the work reported in this paper.

## Data Availability

No data was used for the research described in the article.
