# Supplementary material for: Autoantibodies in the diagnostics, prognostics and follow-up of primary biliary cholangitis
Source: J Transl Autoimmun. 2026 Mar 19;12:100366. doi: 10.1016/j.jtauto.2026.100366 (PMC13068664; doi:10.1016/j.jtauto.2026.100366)
Supplement: Multimedia component 1 [file mmc1.docx]

**Supplementary Table 1**

**Markers involved in the diagnostics, prognostics and therapy monitoring of PBC patients based on current international guidelines**

Markers described in the recommendations of the guidelines or mentioned in the text providing the supporting evidence are presented separately (“in Recommendations” and in “Text”). *R1-R23* are the identifiers of the recommendations in the appropriate guidelines.

*biopsy is indicated after serology and extended imaging, in unexplained intrahepatic cholestasis

**biopsy is indicated if biochemistry and/or serology is negative

^§^qualitative binary scores (involving bilirubin, AP, GGT, AST, albumin, PTC) – Rochester, Barcelona, Paris-I, Rotterdam, Toronto, Paris-II, Ehime

^$^continuous scoring systems (involving bilirubin, AP, AST/ALT, PTC) – GLOBE and UK-PBC score

^#^UDCA response score (involving bilirubin, AP, AST/ALT)

AP: alkaline phosphatase, GGT: gamma-glutamyl transferase, AMA: anti-mitochondrial antibody, ANA: anti-nuclear antibody, AST: aspartate aminotransferase, ALT: alanine aminotransferase, PTC: platelet count, INR: international normalized ratio, anti-HK1: anti-hexokinase 1 antibody, anti-KLHL12: anti-Kelch-like protein 12 antibody, ACA: anti-centromere antibody, anti-PML: anti-promyelocytic leukemia protein antibody, anti-SUMO: anti-small ubiquitin-like modifier antibody, anti-LBR: anti-lamin B receptor antibody, MELD: Model for End-Stage Liver Disease, ELF: Enhanced Liver Fibrosis (score), APRI: AST to Platelet Ratio Index, TG: triglyceride.
